# Implicit theories of women preschool pre-service teachers and emotional intelligence

**DOI:** 10.3389/fpsyg.2023.1260209

**Published:** 2023-11-15

**Authors:** María José Gutiérrez-Cobo, Rosario Cabello, Pablo Fernández-Berrocal

**Affiliations:** ^1^Department of Developmental and Educational Psychology, Faculty of Psychology, University of Málaga, Málaga, Spain; ^2^Department of Basic Psychology, Faculty of Psychology, University of Málaga, Málaga, Spain

**Keywords:** emotional intelligence, MSCEIT, TMMS, implicit theories of intelligence, implicit theories of emotional intelligence, pre-service teacher

## Abstract

**Introduction:**

Pre-service teachers should be prepared to face the emotionally demanding situations associated with the profession. The previous literature suggests that two variables are important for managing teaching demands: emotional intelligence (EI) or the ability to perceive, facilitate, understand, and manage emotions and implicit theories (ITs). ITs refer to the beliefs about the malleability of various life domains. Individuals can be divided into incremental theorists (believing that attributes are malleable) and entity theorists (attributes are fixed).

**Objective:**

This study aimed to evaluate the influence of ITs of intelligence and EI on self-report and ability EI in a sample of female preschool pre-service teachers.

**Method:**

In total, 224 participants (*M* = 21.27, SD = 4.72) were assessed on ability EI (performance and self-report instruments), ITs of intelligence, ITs of EI, age, and parental education.

**Results:**

In our sample, incremental EI—but not intelligence—theories predicted higher scores on self-report and ability EI. In particular, being an incremental theorist of EI predicted 11 and 20% of the variance of the global EI and the managing branch of the ability EI, respectively.

**Conclusion:**

Our findings suggest the importance of ITs of EI for pre-service teachers’ emotional intelligence and open the door to implementing ITs of EI training in this population. These theoretical and practical implications are discussed.

## 1. Introduction

Teaching is a highly emotionally demanding profession in which high levels of stress may lead to psychological disorders and reduced job satisfaction affecting emotional labor (Brackett et al., 2010; Moore, 2012; Keller et al., 2014; Schonfeld et al., 2017). Different variables may protect against these consequences. In the present study, we focus on emotional intelligence (EI) and implicit theories (ITs). Specifically, we aimed to analyze the influence of ITs on EI scores.

Following the model of Mayer and Salovey (1997), EI is “the ability to perceive accurately, appraise, and express emotion; the ability to access and generate feelings when they facilitate thought; the ability to understand emotion and emotional knowledge; and the ability to regulate emotions to promote emotional and intellectual growth” (p. 10). This definition belongs to the ability models of EI. The way EI is evaluated is of theoretical importance since previous studies have revealed low correlations between models and instruments of EI (Goldenberg et al., 2006; Webb et al., 2013; Gutiérrez-Cobo et al., 2017). In particular, ability EI can be assessed by performance or self-report measures (Joseph and Newman, 2010). Performance instruments are objective measures since they evaluate EI using a set of emotional problems with correct and incorrect responses, and they are used in accordance with the *performance-based ability model* of EI. Self-reports evaluate individuals’ subjective perception of their EI and are part of the so-called *self-report ability model*. Another way to operationalize EI is by using the *self-report mixed models*, which consider EI as a combination of social and emotional skills (Bar-On, 2000). Thus, while performance-based ability models understand EI as a form of intelligence, the self-report model conceptualizations are closer to personality traits (Van der Linden et al., 2017; Tommasi et al., 2023). EI is considered a protective factor that buffers these emotional negative consequences in teachers (Yin et al., 2013; Mérida-López and Extremera, 2017; Castillo-Gualda et al., 2019; Pan et al., 2022; Wang and Wang, 2022; Deng et al., 2023) and pre-service teachers (Palomera et al., 2008; Vesely et al., 2014).

ITs refer to the beliefs about the malleability of various life domains such as intelligence, emotions, and EI, among others (Dweck et al., 1995; Dweck, 2012). Concerning these beliefs, we can categorize individuals as entity theorists or incremental theorists. The former believes that a particular attribute is fixed and unable to be modified, while the latter assumes this to be malleable and developed with training and, therefore, subject to control.

Our ITs influence both our cognition and behavior. In the general population, previous studies about IT of intelligence (ITI) have found incremental theorists to show better academic and professional achievement, intrinsic motivation, stronger learning goals, higher tendencies toward making an effort, and better mental health and wellbeing (Robins and Pals, 2002; Blackwell et al., 2007; Dweck, 2012; Schleider et al., 2015; Costa and Faria, 2018; Liu, 2021). In addition, ITI affects brain activation and cognitive processes such as attention or memory (Mangels et al., 2006; Schroder et al., 2014). Focusing on teachers, Stockinger et al. (2021) showed that in a university teacher population, those with a tendency toward incremental theory showed higher teaching quality, as measured by their teaching goals. A study by Tao et al. (2021), in a sample of secondary school teachers, found that entity theorists had higher levels of stress mediated by the interpretation of student failure as immutable.

Individuals also differ in the way they perceive the malleability of EI, that is, in their IT of EI (ITEI). ITEI has been scarcely studied, but a related starting point could be to look at IT about emotions. Being an entity theorist of emotions has been linked to lower social support, less adaptative emotional regulation abilities, and reduced wellbeing; the latter being demonstrated not only in the general population but also in pre-service teachers (Tamir et al., 2007; King and Dela Rosa, 2019; De France and Hollenstein, 2021).

Regarding ITEI, Cabello and Fernández-Berrocal (2015a) analyzed the influence of ITEI on measures within the performance-based ability model of EI in an adult sample. They showed how incremental theorists had higher levels of EI total score. In the school context, we found two studies analyzing the relationships between adolescents’ ITI and ITEI with ability EI (self-report and performance models) and also emotions toward school and academic achievement. In the first study, Costa and Faria (2020a) showed ITEI to be positively related to positive emotions toward school, the understanding branch of the performance-based ability model and the self-report ability model of EI but inversely related to negative emotions toward school. Contrary to expectations, they found a negative and small relationship between ITI and the understanding branch of the performance test of EI. The second study by Costa and Faria (2020b) found a positive relationship between ITEI and academic achievement via two emotion regulation strategies (cognitive reappraisal and emotional suppression). While these three studies constitute a starting point toward studying the influence of ITI and ITEI on EI, no studies have been conducted on teachers or pre-service teachers.

Preschool is a benchmark for young learners as it implies the beginning of the “school age.” This is an important period in which teachers’ EI acts as an early model for emotional management (Poulou, 2017). Therefore, in light of the previous literature, the main objective of the present study was to evaluate the influence of ITI and ITEI on the performance and self-report measures of EI of female preschool pre-service teachers after controlling for relevant sociodemographic variables. Specifically, we included age and parental educational levels given that previous studies have highlighted these factors as influential for EI (Harrod and Scheer, 2005; Fernández-Berrocal et al., 2012; Cabello et al., 2016). Previous literature has pointed to ITs as an important variable that influences human behaviors. However, little is known about its relationship with EI, especially among pre-service teachers. The results could guide the development of future interventions within this population. According to previous results, we proposed the following hypotheses:

- *H1*. A weak relationship will be found between the self-report ability model and the performance-based ability model of EI.
- *H2*. ITEI will have a stronger influence than ITI on both models of EI, after controlling for sociodemographic variables.
- *H3*. Incremental theorists of EI will have higher EI scores according to the performance-based ability model, after controlling for sociodemographic variables.
- *H4*. Incremental theorists of EI will have higher EI scores according to the self-report ability model, after controlling for sociodemographic variables.

## 2. Materials and methods

### 2.1. Participants

The sample consisted of 224 female pre-service preschool teachers from the University of Málaga and Granada, Spain, ranging from 18 to 50 years of age (*M* = 21.27, SD = 4.72). All the participants were Spanish speakers. They took part in the study in exchange for course credits. All participants provided written informed consent after being told of the details of the study. The study was carried out in accordance with the Declaration of Helsinki (World Medical Association, 2008) and was approved by the Research Ethics Committee of the University of Malaga. To be included in the study, participants had to meet the following criteria: (1) being pre-service teachers in preschool education and (2) being Spanish speakers.

### 2.2. Instruments

#### 2.2.1. Implicit theories of intelligence (ITI)

Implicit theories about the malleability of *intelligence* were evaluated using the original scale developed by Dweck (1999). The scale comprises two statements assessing incremental theories (“No matter how much intelligence you have, you can always change it quite a bit” and “You can always significantly change how intelligent you are”) and two that assess entity theories (“You can learn new things, but you cannot really change your basic intelligence” and “Your intelligence is something about you that you cannot change very much”). Participants were asked to report their agreement on a 7-point Likert scale. Entity statements were reverse-scored such that higher scores reflected a more incremental theory of intelligence. The Spanish translation of the ITI instrument was created using a back-translation procedure involving two independent translators, both of whom have PhDs in psychology and who are experts in the topic. In the present sample, Cronbach’s alpha was 0.72.

#### 2.2.2. Implicit theories of EI (ITEI)

This is an adapted version of the scale originally developed by Dweck (1999). This scale evaluates implicit theories about the malleability of the EI concept. It is composed of four items, two of which assess incremental theories (e.g., “You can always significantly change how emotionally intelligent you are”) and another two that evaluate entity theories (e.g., “Your emotional intelligence is something about you that you cannot change very much”). Participants were asked to report on a Likert scale ranging from 1 (strongly disagree) to 7 (strongly agree). In the present sample, Cronbach’s alpha was 0.70.

#### 2.2.3. Mayer-Salovey-Caruso Emotional Intelligence Test

Mayer-Salovey-Caruso Emotional Intelligence Test (MSCEIT, v. 2.0; Mayer et al., 2002; Extremera and Fernández-Berrocal, 2009). The performance-based ability model of EI was used to measure EI through the Spanish version of the MSCEIT. This instrument is composed of 141 items providing a score for each of the four branches of the model (perceiving, facilitating, understanding, and managing) together with a total score. Perceiving emotions is the ability to perceive emotions in oneself, other people, and stimuli; facilitating emotions refers to the ability to use emotions to facilitate cognitive processes; understanding emotions is the ability to understand emotional information (e.g., how emotions progress or combine); and, finally, managing emotions refers to the ability to regulate emotions. Scores are standardized (*M* = 100, SD = 15) with a split-half reliability of 0.93, and the test–retest reliability of the total MSCEIT of 0.86 after 3 weeks (Brackett and Mayer, 2003).

#### 2.2.4. Trait Meta-Mood scale-24

Trait Meta-Mood scale-24 (TMMS-24; Salovey et al., 1995; Fernández-Berrocal et al., 2004). The self-report ability model was used to measure EI through the Spanish version of the TMMS-24. This instrument comprises 24 items that participants have to rate on a Likert scale ranging from 1 (strongly disagree) to 5 (strongly agree). It measures three subfactors: attention or the belief that the individual pays attention to their feelings; clarity or the belief about how clearly an individual perceives their emotions; and repair or the belief in the capacity to regulate their emotions. The Spanish version of the TMMS has shown high internal consistency (Cronbach’s alpha for attention = 0.90, clarity = 0.90, and repair = 0.86) and a satisfactory test–retest reliability (*r* values from 0.60 to 0.83).

#### 2.2.5. Paternal and maternal educational status

Participants were asked about their father’s and mother’s level of education through a self-report question with three response categories: primary studies, high school, and college education.

### 2.3. Procedure

Participants completed the questionnaires through an online platform (LimeSurvey) provided by the university, where blank responses were not allowed in order to avoid missing data.

### 2.4. Data analyses

Descriptive statistics, Pearson’s correlations, and regression analyses were carried out using SPSS version 24.0 (IBM Corporation, Armonk, NY, USA). First, descriptive analyses and Pearson’s correlations were computed for the study variables. Second, stepwise multiple regressions were run to test whether implicit theories of intelligence and EI were a significant predictor of the perceived and ability EI of female pre-service teachers while controlling for age and the parental education status. Five regression models were run to test the association between implicit theories of intelligence and EI and MSCEIT: total (Model 1), perceiving (Model 2), facilitating (Model 3), understanding (Model 4), and managing (Model 5). Additionally, three regression models were run to test the association between implicit theories of intelligence and EI and TMMS: attention (Model 6), clarity (Model 7), and repair (Model 8). The control variables of age and parental education status were included as predictors in each model.

## 3. Results

Descriptive statistics and Pearson’s correlations of the study variables are displayed in Table 1. The descriptive statistics of the variables are within the normal range of values reported in other studies. Pearson’s correlations revealed a positive relationship between ITI and MSCEIT total (*r* = 0.17, *p* < 0.01) and MSCEIT managing (*r* = 0.35, *p* < 0.01), and ITEI and MSCEIT total (*r* = 0.33, *p* < 0.01), MSCEIT managing (*r* = 0.45, *p* < 0.01), MSCEIT understanding (*r* = 0.22, *p* < 0.01), and MSCEIT facilitating (*r* = 0.14, *p* < 0.05). Furthermore, concerning TMMS, we found a positive relationship between ITI and attention (*r* = 0.11, *p* < 0.05), clarity (*r* = 0.16, *p* < 0.01) and repair (*r* = 0.14, *p* < 0.05) and between ITEI and attention (*r* = 0.14, *p* < 0.05) and clarity (*r* = 0.19, *p* < 0.01). In addition, ITI was positively associated with the ITEI (*r* = 0.54, *p* < 0.01). Finally, regarding the relationship between MSCEIT and TMMS, we found only a positive relationship between MSCEIT total and clarity (*r* = 0.13, *p* < 0.05) and between MSCEIT managing and clarity (*r* = 0.18, *p* < 0.01).

**Table 1:** Descriptive statistics (mean [M], standard deviation [SD], skewness [Skew], and kurtosis [Kurt]), and Pearson’s correlation matrix for the study variables.

	M	SD	Skew	Kurt	1	2	3	4	5	6	7	8	9	10	11	12	13
(1) Age	21.27	4.72	4.29	21.39	—												
(2) Mother’s education	2.58	0.87	0.51	−0.88	−0.24^**^	—											
(3) Father’s education	2.44	0.84	0.68	−0.37	−0.14^*^	0.55^**^	—										
(4) Implicit intelligence	21.12	3.67	−0.20	−0.11	0.14^*^	−0.10	−0.05	—									
(5) Implicit EI	22.45	3.53	−0.41	−0.32	0.11^*^	0.03	0.05	0.54^**^	—								
(6) MSCEIT total	103.99	8.31	−0.97	1.35	−0.01	−0.09	−0.11^*^	0.17^**^	0.33^**^	—							
(7) MSCEIT perceiving	107.50	9.73	−1.29	2.42	0.04	−0.18^**^	−0.22^**^	0.03	0.03	0.61^**^	—						
(8) MSCEIT facilitating	96.04	9.61	−0.82	1.08	−0.04	−0.10	−10	0.03	0.14^*^	0.69^**^	0.30^**^	—					
(9) MSCEIT understanding	101.61	9.89	−0.54	0.70	−0.10	0.06	0.02	0.01	0.22^**^	0.52^**^	−0.01	0.13^*^	—				
(10) MSCEIT managing	106.81	13.98	−1.32	2.00	0.07	0.01	0.04	0.35^**^	0.45^**^	0.70^**^	0.13^*^	0.39^**^	0.25^**^	—			
(11) Attention (TMMS)	26.66	5.74	−0.20	−0.28	0.09	0.09	0.04	0.11^*^	0.14^*^	0.04	−0.01	−0.02	0.03	0.11	—		
(12) Clarity (TMMS)	24.46	6.34	0.10	−0.49	0.25^**^	0.04	0.03	0.16^**^	0.19^**^	0.13^*^	0.09	−0.03	0.06	0.18^**^	0.42^**^	—	
(13) Repair (TMMS)	25.06	6.08	0.24	−0.39	0.09	−0.03	0.10	0.14^*^	0.09	0.06	0.05	−0.04	0.03	0.09	0.33^**^	0.36^**^	—

^*^p < 0.05, ^**^p < 0.01.

The results of five linear stepwise regressions to test the association between ITI, ITEI, and MSCEIT total and the four branches are reported in Table 2. In four of the five models analyzed (Models 1, 3, 4, and 5), ITEI significantly predicted the MSCEIT scores beyond the significant contributions of ITI and control variables. Specifically, a greater tendency to adopt incremental theories of EI predicted higher scores on MSCEIT total and three of the four branches: MSCEIT managing, MSCEIT understanding, and MSCEIT facilitating. The unique contribution of ITEI was relatively small for MSCEIT facilitating (*R*^2^ = 0.02) and MSCEIT understanding (*R*^2^ = 0.04) but was the greatest for MSCEIT total (*R*^2^ = 0.11) and particularly MSCEIT managing (*R*^2^ = 0.20). In contrast, the best predictor for MSCEIT perceiving (Model 2) was parental education status, with a small unique contribution (*R*^2^ = 0.04).

**Table 2:** Stepwise regression models predicting ability EI (MSCEIT total and four branches) and perceived EI (TMMS) from implicit theories about intelligence and EI and controlling for the variables of age and the father and mother level of education (*N* = 224).

	Variable	B	*β*	*p*	DF	F	*p*	*R*^2^Adj	Delta *R*^2^
Model 1 (MSCEIT total)									
Step 1					1, 222	27.73	< 0.001	0.11	
	Implicit EI	0.78	0.33	< 0.001					
Step 2					2, 221	16.09	0.05	0.12	0.01
	Implicit EI	0.80	0.34	< 0.001					
	Father’s education	−1.26	−0.13	0.05					
Model 2 (MSCEIT perceiving)									
Step 1					1, 222	10.79	0.001	0.04	
	Father’s education	−2.49	−0.22	0.001					
Model 3 (MSCEIT facilitating)									
Step 1					1, 222	4.40	0.04	0.02	
	Implicit EI	0.38	0.14	0.04					
Model 4 (MSCEIT understanding)									
Step 1					1, 222	10.80	0.001	0.04	
	Implicit EI	0.60	0.22	0.001					
Model 5 (MSCEIT managing)									
Step 1					1, 222	56.98	< 0.001	0.20	
	Implicit EI	1.79	0.45	< 0.001					
Step 2					2, 221	31.01	< 0.001	0.21	0.01
	Implicit EI	1.48	0.38	< 0.001					
	Implicit intelligence	0.55	0.15	0.041					
Model 6 (TMMS: Attention)									
Step 1					1, 222	4.22	0.04	0.01	
	Implicit EI	0.22	0.14	0.04					
Model 7 (TMMS: Clarity)									
Step 1					1, 222	15.27	< 0.001	0.06	
	Age	0.34	0.254	< 0.001					
Step 2					2, 221	10.90	< 0.001	0.08	0.02
	Age	0.32	0.24	< 0.001					
	Implicit EI	0.29	0.16	0.01					
Model 8 (TMMS: Repair)									
Step 1					1, 222	4.73	0.03	0.02	
	Implicit intelligence	0.24	0.14	0.03					

Implicit intelligence, implicit theories about intelligence; Implicit EI, implicit theories about emotional intelligence.

The results of three linear stepwise regressions testing the association between ITI, ITEI, and TMMS dimensions are reported in Table 2. In two models (Models 6 and 7), ITEI significantly predicted the TMMS beyond the significant contributions of ITI and control variables. A greater tendency toward incremental theories of EI predicted higher scores on attention and clarity although its unique contribution was relatively small for attention (*R*^2^ = 0.01) and clarity (*R*^2^ = 0.02). In contrast, the best predictor for repair (Model 8) was ITI with a small contribution (*R*^2^ = 0.02).

## 4. Discussion

The present study aimed to analyze the influence of ITI and ITEI on EI. Specifically, we wanted to determine to what extent incremental or entity theories of intelligence and EI impact the EI ability, assessed through both objective performance and subjective self-report measures.

Starting with our target variables, and as expected (H1: A weak relationship would be found between the self-report ability model and the performance-based ability model of EI), we only found weak relationships between some of the scales of the performances and the self-report ability models of EI. Specifically, we only found significant positive correlations between the clarity subscale of the TMMS and the total and the understanding branch of the MSCEIT. This is congruous with previous literature and emphasizes that although both models are conceptualized in the same way, they do not cover the same construct (Goldenberg et al., 2006; Webb et al., 2013; Gutiérrez-Cobo et al., 2017). Therefore, it is important to consider this result to understand the remaining outcomes.

Concerning the main aim of our study, that is, analyzing the influence of ITI and ITEI on EI scores (via both self-report and performance measures), we hypothesized (H2) that ITEI would have a stronger impact than ITI on these variables. Correlational analyses revealed that ITI was positively and weakly related to both the total and managing branches of the performance-based ability model of EI and the three subscales of the EI self-report ability model. ITEI correlated positively and weakly with the total and the facilitating and understanding branches of the MSCEIT and moderately with the managing branch, that is, with the performance-based ability model. In addition, weak positive correlations were found between ITEI and the attention and clarity subscales of the TMMS or the self-report ability model. To determine the variable that more strongly influences EI, eight multiple regression analyses were carried out. These analyses showed that ITEI predicted the total and the managing, facilitating, and understanding branches of the MSCEIT and the attention and clarity subscales of the TMMS beyond the control variables and, more importantly, beyond ITI. Only the repair subscale of the TMMS was better predicted by the ITI. This indicates that, as hypothesized, being an incremental theorist of EI influences the EI of the pre-service teachers to a greater extent than believing intelligence is malleable. Overall, this result is consistent with those of previous studies with adolescents and the general population (Cabello and Fernández-Berrocal, 2015a; Costa and Faria, 2020a,b). These findings highlight the domain-specificity of IT measures, with the ITEI (but not ITI) having the greatest impact on EI (Romero et al., 2014; Costa and Faria, 2020a).

The present findings also support our H3, that is, incremental theorists of EI showed higher scores on the performance-based ability model of EI. This was predicted for all the EI branches except the perceiving branch of the MSCEIT. Nonetheless, this result was particularly significant for both the total and managing branches of EI, for which ITEI explained 11 and 20% of the variance, respectively. This result is consistent with the findings of Cabello and Fernández-Berrocal (2015a) which also suggests that being an incremental theorist was associated with higher scores of the total EI. Regarding the self-report model of EI and supporting H4 (incremental theorists of EI will have higher EI scores according to the self-report ability model, after controlling for sociodemographic variables), ITEI was also a predictor of this measure (although it explained no more than 2% of the variance) and the attention and clarity subscales.

Therefore, taken together, the present results suggest that ITEI had a greater influence on the performance EI scores than the self-perceptions of the pre-service teachers, and principally, this influence is on the ability to regulate emotions. It is particularly interesting to note that the managing branch was most influenced by ITEI, given that the ability to regulate emotions is of central importance for creating adaptive responses in emotional situations (Megías-Robles et al., 2019). Furthermore, the employment of adaptive strategies to regulate emotions plays a critical role in personal and social success, as well as in wellbeing (Côté, 2014; Cabello and Fernández-Berrocal, 2015b; Balzarotti et al., 2016).

The lower influence of ITEI on self-perceived EI compared with performance measures is inconsistent with previous studies where the opposite pattern of results has been reported (Costa and Faria, 2020a,b). However, there are two possible explanations for this result. First, these previous studies employed a performance test that only evaluated one of the four branches of the EI model—the understanding ability—and found that ITEI had little impact on this ability. In the present study, the influence of ITEI on the understanding branch is also very low (4%), while the managing branch was most strongly predicted by ITEI (20%). Second, regarding the weak influence of ITEI on self-report EI in our sample compared to Costa and Faria (2020a,b), it is important to consider that our sample comprised only female adults. Previous studies have shown how women tend to underestimate their EI when evaluated with self-report measures, which could have influenced the results (Petrides and Furnham, 2000; Côté, 2014).

This study has some limitations. First, the correlational nature of the study prevents us from making any causal inferences. Thus, future investigations should focus on causally analyzing the impact of ITEI on both the performance and self-report EI scores by, for instance, testing the efficacy of ITEI programs. Second, our sample comprised only female adults, and thus, the results cannot be generalized to male adults. The recruitment of our sample reflected the higher proportion of female students who were enrolled in the teacher training courses. Finally, only 17 male adults were included in our sample, and we decided to remove them from the analysis since they only accounted for 7% of the sample, leading to underpowered analyses (Anderson, 2021). However, future research should include a male sample to increase the generalizability of the findings, particularly given the gender differences in EI reported in previous studies (Cabello et al., 2016; Vega-Hernández et al., 2017).

## 5. Conclusion

In conclusion, in a sample of female pre-service teachers, ITEI appears to be associated with managing emotions (which is the most complex branch of the EI model) while showing a weaker relationship with self-perceived EI. These results have important theoretical and clinical implications. Specifically, our results contribute to the literature on ITs by suggesting the importance of ITEI for the EI of pre-service teachers. This also opens the door to new research studies in teachers and pre-service teachers of other grades (e.g., primary and university). Finally, our results are of clinical relevance since they could inform the development and implementation of ITEI training programs in order to evaluate their impact on the EI of female pre-service teachers. Such training could be included in the career curriculum.

## Data availability statement

The original contributions presented in the study are publicly available. This data can be found here: https://riuma.uma.es/xmlui/handle/10630/22905.

## Ethics statement

The studies involving humans were approved by the Research Ethics Committee of the University of Malaga. The studies were conducted in accordance with the local legislation and institutional requirements. The participants provided their written informed consent to participate in this study.

## Author contributions

MG-C: Conceptualization, Data curation, Funding acquisition, Investigation, Methodology, Resources, Validation, Writing – original draft. RC: Conceptualization, Formal analysis, Funding acquisition, Investigation, Writing – review & editing. PF-B: Conceptualization, Formal analysis, Funding acquisition, Investigation, Methodology, Project administration, Supervision, Writing – review & editing.
